# Electronic Health Record-Based Machine Learning Model for Predicting Disease Activity in Patients with Rheumatoid Arthritis

**DOI:** 10.34133/hds.0461

**Published:** 2026-06-08

**Authors:** Xiaoying Zhang, Chun Li, Zelin Yun, Yi Zhao, Shengguang Li, Wenqiang Fan, Limin Ma, Xiangheng Meng, Ru Li, Fangmin Xu, Jing Yang, Zhanguo Li

**Affiliations:** ^1^ Department of Rheumatology & Immunology, Peking University People’s Hospital, Beijing, China.; ^2^ Department of Rheumatology and Immunology, Peking University Third Hospital, Beijing, China.; ^3^ Beijing Key Laboratory for Rheumatism Mechanism and Immune Diagnosis (BZ0135), Beijing, China.; ^4^ Department of Rheumatology and Immunology, Xuanwu Hospital Capital Medical University, Beijing, China.; ^5^ Department of Rheumatology and Immunology, Peking University International Hospital, Beijing, China.; ^6^ Department of Rheumatology and Immunology, Xinxiang Central Hospital, Henan, China.; ^7^ Key Laboratory of Universal Wireless Communications, Ministry of Education, Beijing University of Posts and Telecommunications, Beijing, China.; ^8^ Department of Rheumatology and Immunology, Mianyang Central Hospital, Sichuan, China.

## Abstract

**Background:** The use of initial clinical assessments to predict therapeutic outcomes via machine learning (ML) is a promising frontier in precision medicine. The study aims to construct ML models capable of predicting disease activity in patients with rheumatoid arthritis (RA), thereby optimizing clinical decision-making and treatment selection. **Methods:** This multicenter retrospective study analyzed electronic health records (EHRs) from 1,864 patients with RA across 5 tertiary hospitals in China between 2017 and 2022. The dataset from Peking University People’s Hospital (PKUPH) was employed as the training and internal validation cohort, whereas data from 4 other centers were used for external validation. Longitudinal variables, including demographics, laboratory indices, and medication regimens, at baseline, 3-month, and 6-month follow-up were integrated to capture dynamic disease patterns. Four ML models were trained to predict disease status 6 months post-treatment, with the primary outcome defined as clinical remission (disease activity score in 28 joints with erythrocyte sedimentation rate ≤ 2.6). **Results:** The final analysis included 1,629 patients from PKUPH and 235 from 4 other tertiary hospitals. In the internal validation phase, the optimal model achieved an accuracy of 95.3% and an area under the receiver operating characteristic curve (AUROC) of 0.971, with sensitivity, specificity, positive predictive, and negative predictive values of 98.1%, 84.2%, 96.1%, and 91.8%, respectively. The model exhibited generalizability in external validation, presenting an accuracy of 87.3% and an AUROC of 0.922. Furthermore, in the multiclass task of stratifying patients into remission, low, moderate, or high disease activity, the deep neural network model showed an accuracy of 68.6% and AUROC of 0.860. **Conclusions:** Longitudinal clinical data extracted from EHRs can be effectively leveraged to develop prognostic models. This study confirms that deep learning approaches trained on large-scale multicenter cohorts can accurately predict disease trajectories in RA, offering a valuable tool for personalized patient management.

## Introduction

Rheumatoid arthritis (RA) is a common systemic inflammatory autoimmune disease characterized by painful, swollen joints that can severely impair physical functions and quality of life [1,2]. It affects 0.5% to 1% of American adults and 0.28% of people in China [1,3]. Although the research on assessing the efficacy of biological disease-modifying antirheumatic drugs (bDMARDs), conventional synthetic disease-modifying antirheumatic drugs (csDMARDs), and targeted synthetic disease-modifying antirheumatic drugs (tsDMARDs), and the safety of pharmacological treatments has grown rapidly, no markers have been established for a precision medicine approach in clinical practice [4,5]. Initial effective therapy can contribute to disease remission and reduce economic burdens [6,7]. However, physicians often encounter situations where the treatment fails to achieve disease remission or the disease relapses after initial therapy because of patient heterogeneity and the lack of clinical evidence to guide drug choices decisively [8–13]. Therefore, models that can predict therapy responses are essential.

Machine learning (ML), a branch of artificial intelligence (AI), is a computerized analytical technique that applies algorithms to recognize relationships in data and predict outcomes [14–16]. With the improvement in the electronic health records (EHRs) of patients’ demographic features and medical history, ML models can now be trained and evaluated to predict individual treatment responses [17]. Prior researches typically fall into 2 categories. The first category involves models integrating multi-omics data, such as genomics or transcriptomics data, or specific biomarkers, to predict responses to bDMARDs [18,19]. Although these approaches are scientifically compelling, they require expensive assays and invasive sampling, limiting their scalability in routine clinical practice. The second category utilizes standard clinical and demographic variables available in registries [20,21]. However, many studies in this category are restricted by their small sample sizes, single-center designs, or modest predictive performance, and few have been rigorously validated in external cohorts with diverse data distributions [22]. These limitations diminish the suitability of ML models for forecasting outcomes on the basis of EHRs. Therefore, we need a robust, noninvasive ML model that utilizes sequential clinical data across multicenter populations to predict disease activity accurately and guide treatment.

Our study aims to employ ML techniques to establish models that predict treatment efficacy after the baseline evaluation. By using the disease activity score in 28 joints with erythrocyte sedimentation rate (DAS28-ESR) [23] in patients with RA after treatment initiation, we developed and validated 4 ML models using structured EHR data for predicting outcomes after rigorous data collection and processing. External validation across distinct hospitals demonstrated that our models had high efficacy. The findings of our work intend to offer an objective evidence base for clinical decision-making and selecting optimal therapeutic strategies.

## Methods

### Design

This study is a national multicenter cohort investigation performed in China. Its primary objective is to predict the therapy responses of patients with RA by training a supervised ML model using parameters obtained prior to treatment. Therapy responses were categorized as remission (DAS28 ≤ 2.6) and nonremission (DAS28-ESR > 2.6). The performance of the ML models in predicting disease activity [DAS28-ESR > 5.1 for high disease activity (HDA), >3.2 to ≤5.1 for moderate disease activity (MDA), >2.6 to ≤3.2 for low disease activity (LDA), and ≤2.6 for remission] was also tested as the secondary outcome.

### Study population and data sources

Structured EHRs were collected from 5 tertiary hospitals, namely, Peking University People’s Hospital (PKUPH), Xuanwu Hospital of Capital Medical University, Peking University International Hospital, Mianyang Central Hospital, and Xinxiang Central Hospital. All patients met the RA classification criteria of the 1987 American College of Rheumatology (ACR) and 2010 ACR/European League Against Rheumatism (EULAR) criteria [24,25]. The EHRs of 6,729 patients with RA were collected. Specifically, 3,976 EHRs were from PKUPH, and 2,753 EHRs were from other 4 centers. The data collection period was 2017–2022. Adherence to the guidelines of the REporting of studies Conducted using Observational Routinely collected health Data (RECORD) and Transparent Reporting of a multivariable prediction model for Individual Prognosis Or Diagnosis artificial intelligence (TRIPOD-AI) was maintained. The Prediction model Risk Of Bias ASsessment Tool artificial intelligence (PROBAST-AI) should be used to assess the risk of bias [26].

### Datasets

All patients were followed up at each center for at least 6 months. Their demographic, clinical, and serological data were recorded at the baseline, 3-month follow-up, and 6-month follow-up. Demographic features at baseline encompassed age, sex, disease duration, and RA family history. Laboratory variables included C-reactive protein (CRP), erythrocyte sedimentation rate (ESR), rheumatoid factor (RF), and anti-cyclic citrullinated peptide antibody (anti-CCP). Clinical features encompassed 28 tender joint counts (TJC28), 28 swollen joint counts (SJC28), patient global assessment of disease activity (PtGA), physician global assessment of disease activity (PhGA), and DAS28-ESR. Joint deformity and rheumatoid nodules were recorded. Joint radiographic (x-ray) and ultrasonographic findings and chest computed tomography were documented. Detailed pharmaceutical records encompassing treatment duration and prescribed daily dosage were documented for all the participants.

### Therapeutic combination and outcome metric

Drug classifications included csDMARDs, bDMARDs, traditional Chinese medicine, nonsteroidal anti-inflammatory drugs (NSAIDs), and glucocorticoids. Specifically, csDMARDs comprised methotrexate, leflunomide, sulfasalazine, iguratimod, and hydroxychloroquine, and bDMARDs included infliximab, adalimumab, recombinant human tumor necrosis factor receptor–Fc fusion protein, etanercept, and tocilizumab. Traditional Chinese medicine referred to tripterygium wilfordii, total glucosides of white peony, Zhengqingfengtongning. NSAIDs refers to meloxicam, loxoprogen, ibuprofen, naproxen, diclofenac, ketoprofen, piroxicam, oxaprozin, nabumetone, etoricoxib, and celecoxib. Glucocorticoids included prednisone, prednisolone, and methylprednisolone. They received monotherapy or combination therapy on the basis of their medical conditions.

The DAS28-ESR results were grouped into 4 categories: remission (REM, DAS28-ESR ≤ 2.6), LDA (2.6 < DAS28-ESR ≤ 3.2), MDA (3.2 < DAS28-ESR ≤ 5.1), and HDA (DAS28-ESR > 5.1) [27]. These categories were further consolidated into binary disease activity states: remission (DAS28 ≤ 2.6) or nonremission (LDA, MDA, or HDA; DAS28-ESR > 2.6).

### Data preprocessing

Data preprocessing comprised 2 steps: data integration and quantification. Feature augmentation and data balance were adopted to exploit EHRs efficiently.

In the first step, data, including demographics, clinical variables, and medications, were extracted from EHRs. Irrelevant features (e.g., patients’ city or income) were excluded. Patient data were cleaned when demographic characteristics and any parameter required for calculating DAS28-ESR were unavailable. More than 3 instances of missing values in features (RF, anti-CCP, ESR, and CRP) also resulted in data cleaning. As a result, the EHRs of 1,864 patients with RA were included in data processing.

The proportions of missing data were 1.17% for ESR, 15.58% for CRP, 16.42% for RF, and 53.2% for ACPAs (anti-citrullinated protein antibodies). The overall proportion of missing data was 21.59% (Table S1). The comparison of baseline characteristics, including ESR, CRP, RF, and anti-CCP, between patients with and without missing data revealed no significant differences, suggesting that systematic bias is unlikely (*P* = 0.228, *P* = 0.123, *P* = 0.816, and *P* = 0.589, respectively). Mean imputation was employed for missing data, and the average value of known detection values was used to fill the gaps [28]. A sensitivity analysis was conducted with mean imputation, median imputation, *k*-nearest neighbors (KNNs), and multiple imputation (IterativeImpuer) [29,30]. Due to high missingness of ACPA, further analysis was conducted based on 3 categories, namely, positive ACPA, negative ACPA, and missing. Using various imputation methods, the model accuracy was consistently 0.8672 (Table S2).

The second step involved data quantification. The initial DAS28-ESR results were transformed into a binary disease activity state, as described above. The all *k*-nearest neighbor (ALLKNN) algorithm was applied after train/test split to exert an undersampling effect and address the unbalanced data distribution [31].

### Model construction and validation

**Fig. 1.**
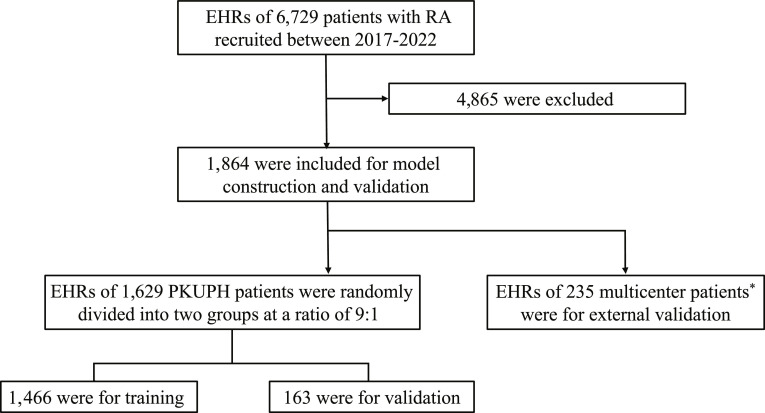
Flowchart of the study profile. *EHRs of multicenter patients were from Xuanwu Hospital of Capital Medical University, Peking University International Hospital, Mianyang Central Hospital, and Xinxiang Central Hospital. EHR, electronic health record; PKHPH, Peking University People’s Hospital; RA, rheumatoid arthritis

After data processing, the charts of 1,629 patients at PKUPH were randomly allocated into training and test datasets at a ratio of 9:1. The prediction model was developed by employing various ML algorithms, including support vector machine (SVM), adaptive boosting (AdaBoost), random forest (RF), and deep neural network (DNN) (Supplemental Materials). The external validation utilized EHRs from 235 multicenter patients (Fig. 1). The training datasets included data collected at baseline, 3 months, and 6 months. The predictive model was developed using baseline and 3-month data as input features to predict the 6-month outcome.

The final parameters of the prognostic model were determined by balancing sensitivity, specificity, positive predictive value (PPV), and negative predictive value (NPV). Accuracy (consistency rate) and the area under the receiver operating characteristic curve (AUROC) were used to assess the efficacy of the predictive model. The specific application models could be accessed at http://101.200.191.108:80.

### Model interpretation and feature importance

SHapley Additive exPlanations (SHAP) analysis was applied to assess the overall feature importance in the ML models [32]. An appropriate SHAP explainer was used based on model type: DeepExplainer for DNN, KernelExplainer for SVM and AdaBoost, and TreeExplainer for RF.

### Statistical analysis

Scikit-Learn version 0.24.2 based on Python version 3.8 was used to construct the SVM, AdaBoost, and RF models, and Keras was employed for DNN. The dataset was first divided into *K* subsets to select model parameters by using the *K*-fold cross-validation method. Sequentially, one subset was designated as the validation set, whereas the remaining *K* − 1 subset served as the training set. This process was repeated *K* times, with each iteration employing a different validation subset. The parameter set yielding the highest accuracy was ultimately selected as the final model parameters. *K*-fold cross-validation ensured the full utilization of data and provided reliable model performance assessment. In this study, *K* = 10 was set to enhance stability. The specific parameters are given in detail in Table S3. IBM SPSS Statistics software version 26.0 was applied to process all descriptive statistics. Quantitative variables with asymmetrical distributions were analyzed by using the Mann–Whitney *U* test, and differences between groups were assessed with the chi-square test for categorical variables. Significant differences were defined as *P* < 0.05 (2-sided *P* for the significance test).

## Results

### Study population

Significant heterogeneity was observed between the training and validation cohorts. Table 1 shows that the baseline DAS28-ESR from PKUPH was 5.4 (4.8, 6.2) and that from the other centers was 6.1 (5.5, 6.7). We found significant differences in disease duration, TJC28, SJC28, PtGA, PhGA, RF, anti-CCP, ESR, and CRP between the PKUPH and multicenter cohorts. Baseline DAS28-ESR was consistently identified as the dominant predictor across algorithms. As illustrated in the feature importance calculated by the Gini importance shown in Fig. S1, baseline DAS28-ESR was the most crucial feature for the DNN, AdaBoost, and RF models, and the second-most important for the SVM model.

**Table 1.** The baseline characteristics of the study population

Characteristic	PKUPH (*n* = 1,629)	Multicenter cohort ^b^ (*n* = 235)	*P* value
Age, years	51 (43, 59)	49 (40, 60)	0.154
Female, *n* (%)	1,353 (83.1)	197 (84.5)	0.764
RA duration, months	68.2 (25.8, 128.4)	48 (12.0, 108.0)	<0.001 ^a^
Clinical evaluations			
DAS28-ESR	5.4 (4.8, 6.2)	6.1 (5.5, 6.7)	<0.001 ^a^
TJC28	12 (8, 19)	10 (7, 16)	<0.001 ^a^
SJC28	7 (5, 12)	6 (4, 10)	<0.001 ^a^
PtGA	60 (50, 70)	70 (60, 75)	0.036 ^a^
Laboratory tests			
ESR, mm/h	42.0 (27.0, 63.0)	48.0 (31.0, 72.0)	0.004 ^a^
CRP, mg/l	13.5 (5.6, 28.9)	6.2 (2.0, 18.4)	<0.001 ^a^
RF, IU/ml	125.0 (44.9, 339.5)	91.1 (26.6, 370.0)	<0.001 ^a^
Anti-CCP, IU/ml	193.0 (56.0, 411.1)	100.0 (33.7, 242.8)	0.003 ^a^

Anti-CCP, anti-cyclic citrullinated peptide; CRP, C-reactive protein; DAS28, disease activity score using 28 joint counts; ESR, erythrocyte sedimentation rate; PtGA, patient global assessment of disease activity; PhGA, physician global assessment of disease activity; PKHPH, Peking University People’s Hospital; RA, rheumatoid arthritis; RF, rheumatoid factor; SJC28, swollen joint count (of 28); TJC28, tender joint count (of 28)

^a^ Multicenter included Xuanwu Hospital of Capital Medical University, Peking University International Hospital, Mianyang Central Hospital, and Xinxiang Central Hospital.

^b^ P < 0.05.

### Model performance on training and validation cohorts

**Fig. 2.**
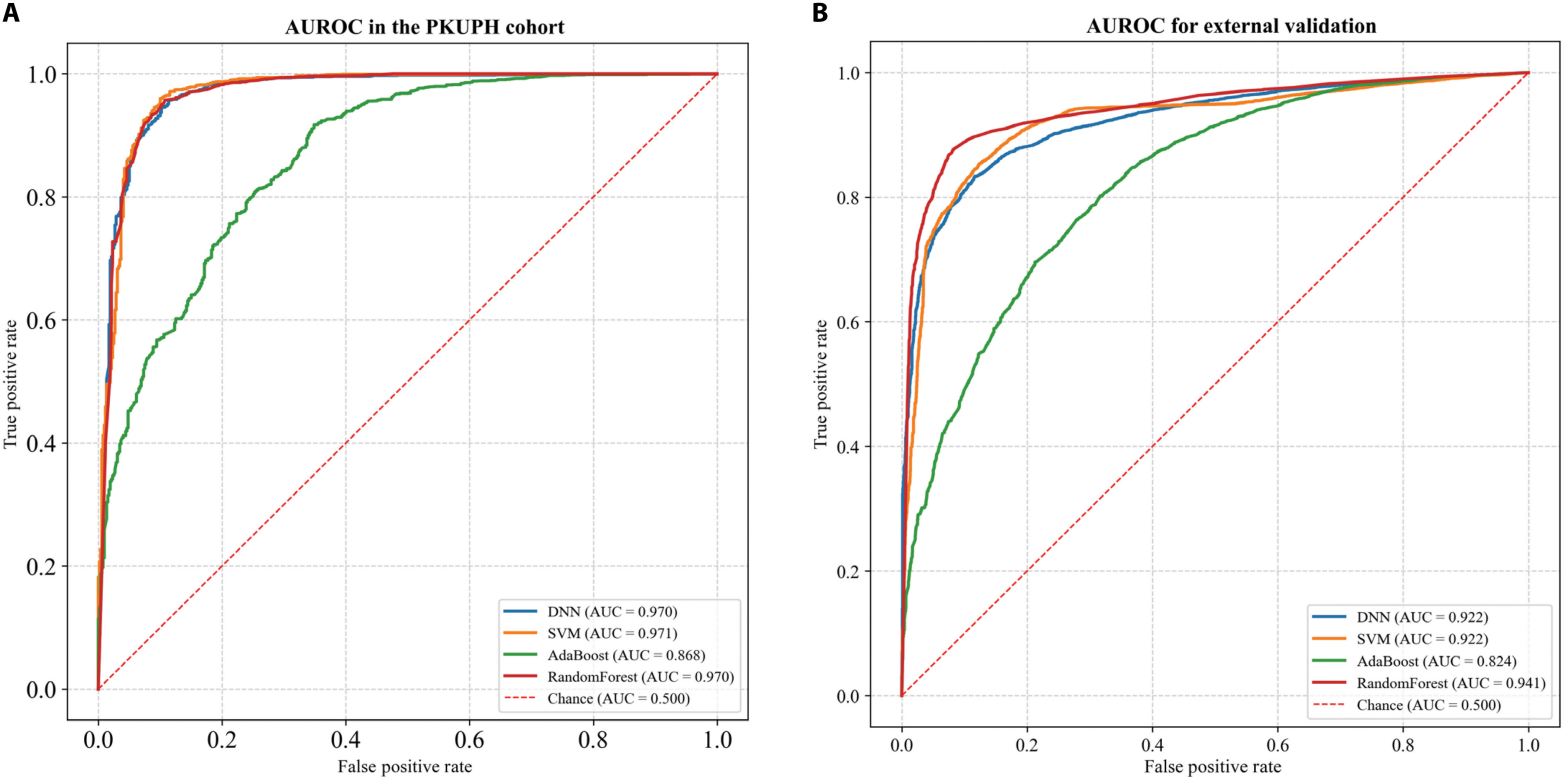
Evaluation of predictive ML models using AUROC. (A) AUROC in the PKUPH cohort. (B) AUROC for external validation. Different colors were used to represent ML models. AdaBoost, adaptive boosting; AUROC, area under the receiver operating characteristic curve; DNN, deep neural network; PKUPH, Peking University People’s Hospital; SVM, support vector machine.

**Fig. 3.**
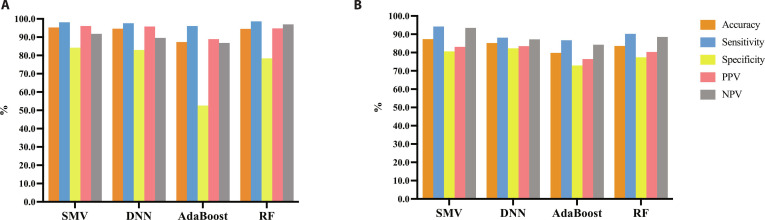
The accuracy of different ML models. (A) Performance of models in the PKUPH cohort and (B) for external validation. Different colors were used to represent ML models. AdaBoost, adaptive boosting; DNN, deep neural network; NPV, negative predictive value; PPV, positive predictive value; RF, random forest; SVM, support vector machine.

In the internal validation dataset (PKUPH), the ML models achieved high diagnostic accuracy for distinguishing remission from nonremission. The accuracy of each model on the PKUPH validation dataset is shown in Table 2 and Fig. 2A, and the AUROCs of the models are given in Fig. 3A. Notably, among models, the SVM model had the highest performance, achieving an accuracy of 95.3% and an AUROC of 0.971.

**Table 2.** The accuracy of different machine learning models in PKUPH validation. Overall accuracy and other indicators reflecting the efficacy of different machine learning models for primary outcome prediction were displayed.

Model	Accuracy (%)	Sensitivity (%)	Specificity (%)	PPV (%)	NPV (%)
DNN	94.6	97.6	83.0	95.8	89.6
SVM	95.3	98.1	84.2	96.1	91.8
AdaBoost	87.3	96.1	52.6	88.9	86.8
RF	94.5	98.6	78.4	94.8	97.0

AdaBoost, adaptive boosting; DNN, deep neural network; NPV, negative predictive value; PKUPH, Peking University People’s Hospital; PPV, positive predictive value; RF, random forest; SVM, support vector machine

The models maintained robust predictive capability when tested on the external multicenter dataset (Table 3 and Figs. 2B and 3B). The SVM model achieved an accuracy of 87.3% and an AUROC of 0.922 on the external validation cohort. The DNN and RF models demonstrated accuracies of 85.2% and 83.6%, respectively, whereas AdaBoost had an accuracy of 79.8%.

**Table 3.** The accuracy of different machine learning models in external validation. Overall accuracy and other indicators reflecting the efficacy of different machine learning models for primary outcome prediction were displayed.

Model	Accuracy (%)	Sensitivity (%)	Specificity (%)	PPV (%)	NPV (%)
DNN	85.2	88.1	82.3	83.5	87.2
SVM	87.3	94.2	80.6	83.1	93.5
AdaBoost	79.8	86.7	72.9	76.4	84.3
RF	83.6	90.2	77.4	80.3	88.5

AdaBoost, adaptive boosting; DNN, deep neural network; NPV, negative predictive value; PPV, positive predictive value; RF, random forest; SVM, support vector machine

Among the 4 ML models evaluated, SVM had the highest predictive accuracy on the internal and external validation cohorts (Fig. 3). Consequently, the SVM model is recommended because of its consistent performance across distinct datasets.

### Performance in disease activity stratification

Beyond binary classification (remission versus nonremission), we evaluated the models for their ability to stratify patients into 4 specific disease activity states (REM, LDA, MDA, and HDA). In this complex multiclass classification task, the DNN model obtained an accuracy of 68.6% and an AUROC of 0.860 (Table S4 and Fig. S2).

### Visualization of feature importance

**Fig. 4.**
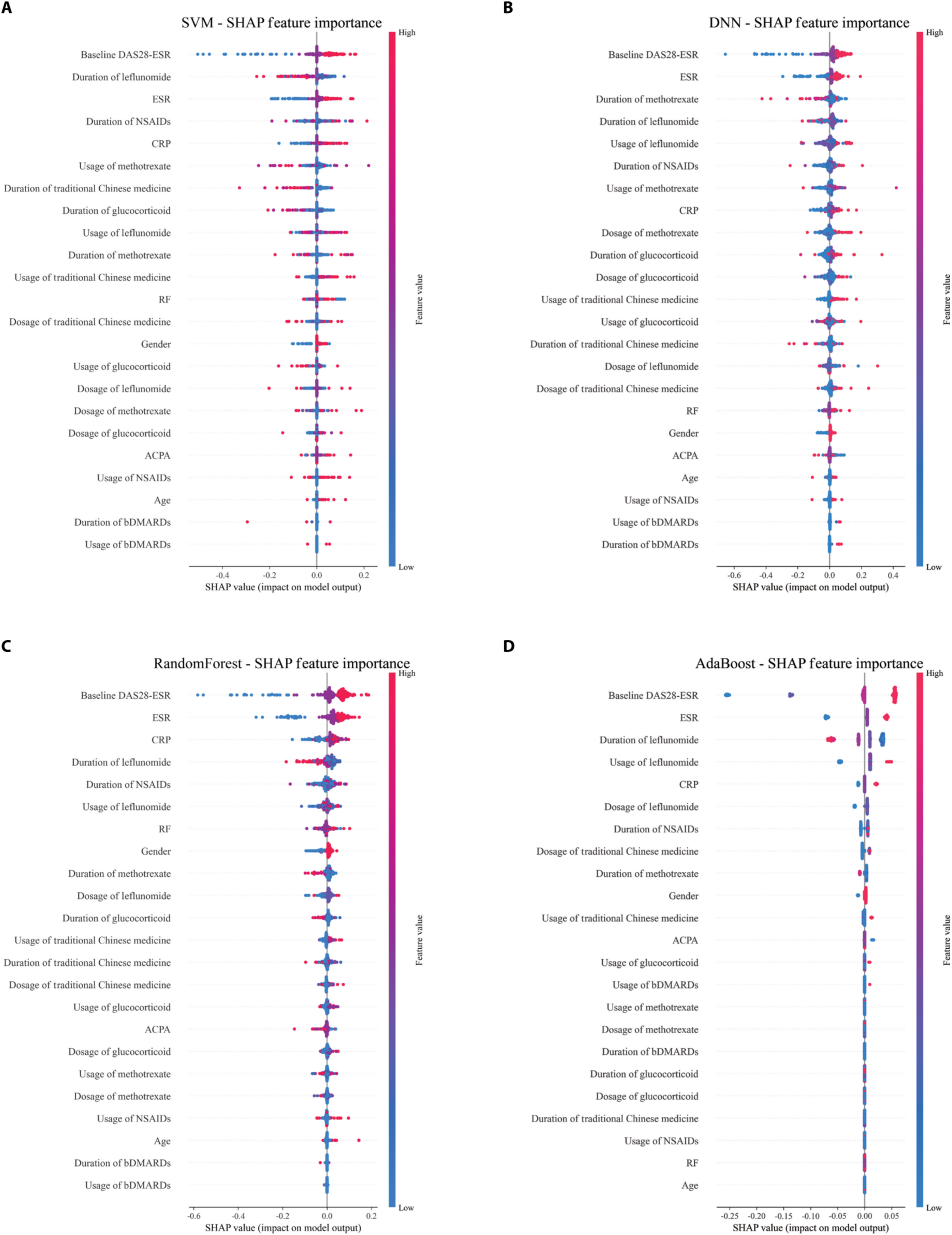
Variable importance in 4 ML models. SVM (A), DNN (B), RF (C), and AdaBoost (D) model.

We performed SHAP analysis to illustrate how the variables contributed to the prediction of 6-month disease activity by the models. The feature ranking on *y* axis indicated the importance features of the model (Fig. 4 and Fig. S3). The SHAP value on the *x* axis was a unified index of its influence on the predicted outcome.

## Discussion

In this study, we developed ML models to predict the disease activity of patients with RA 6 months after treatment initiation. The findings derived from the structured datasets of EHRs indicate that our ML models could effectively predict therapy responses. Notably, the models exhibited robust performance in a highly diverse population, demonstrating resilience against overfitting. Given the marked disparities in baseline features between the PKUPH and multicenter cohorts, we are optimistic about the models’ potential applicability in real-world scenarios.

While a previous study achieved good predictive performance using complex omics data, such approaches are often cost-prohibitive for routine monitoring [33]. Our model relies solely on standard EHR data, making it a cost-effective tool for widespread clinical application. In contrast to models that depend exclusively on baseline characteristics [17], our model incorporated longitudinal data. This dynamic approach allows for the accurate prediction of the 6-month outcome. Previously, the application of deep learning models in RA was primarily confined to image analysis [34,35]. However, our study found that deep learning algorithms can achieve favorable performance in analyzing structured data by using a moderate sample of EHRs. Our finding expands the scope of deep learning algorithms beyond image analysis and lays the foundation for large-scale studies.

Our predictive model offers a convenient and swift means of forecasting disease trajectories and assessing the outcomes of specific drug combinations during the routine clinical visits of individuals with RA. By inputting necessary features, the model (accessible at http://101.200.191.108:80) can efficiently reveal the likely disease course. For example, if our model predicts a low probability of remission at 6 months, then the clinician is alerted to consider intensifying therapy, such as adding a bDMARD.

Traditional models often assume linear relationships between variables and outcomes. However, the progression of RA involves complex, nonlinear interactions between inflammation markers, medication combinations, and patient characteristics. Our results show that ML algorithms can handle this high-dimensional complexity more effectively than traditional methods, which typically yield lower predictive accuracy in similar contexts. While ML offers high predictive power, it is often viewed as a black box. Integrating statistical methods can enhance model interpretability and robustness [36]. We believe that this hybrid approach will be essential for building trust among clinicians and facilitating the adoption of AI tools in precision medicine.

The superior performance of the SVM and DNN algorithms to that of other models in our study can be attributed to their inherent architectural advantages for handling complex, high-dimensional clinical data. SVM excels in scenarios with high-dimensional feature spaces by maximizing the margin between classes, thereby reducing overfitting risk even with moderate sample sizes [37]. Its effectiveness in our cohort likely stems from its robustness against outliers and ability to capture nonlinear relationships through kernel functions. This characteristic is particularly relevant given the heterogeneous nature of RA disease trajectories. The DNN model’s strong performance, particularly in the multiclass classification task, reflects its capacity to learn hierarchical representations and extract complex interactions between longitudinal variables automatically without requiring manual feature engineering [17]. By contrast, AdaBoost’s low performance may be attributed to its sensitivity to noisy data and outliers, which are common in real-world EHR data. Meanwhile RF, despite its robustness, may have been limited by its tendency toward bias in multiclass imbalanced datasets [38,39].

While the SVM model achieved exceptional sensitivity, the clinical consequences of false positives and false negatives need careful consideration. False positives may lead to insufficient therapy intensification, potentially resulting in suboptimal disease control, joint damage progression, and increased long-term disability [40]. Conversely, false negatives could result in unnecessary treatment escalation, exposing patients to elevated risks of adverse events, increased medication costs, and psychological burden [41]. Our model has a high NPV, which suggests that it is reliable for ruling out remission and supports its utility as a screening tool to identify patients requiring close monitoring or alternative therapeutic strategies. However, given the above potential risks, we emphasize that our model should serve as a decision-support tool rather than a replacement for clinical judgment.

We employed SHAP to quantify and visualize feature contributions across different algorithms and thus enhance the interpretability of our predictive models. Notably, the SHAP values for the same feature varied across models. This variation is expected and reflects inherent differences in model architectures, underlying assumptions, and learning mechanisms. These findings highlight the value of incorporating explainability techniques such as SHAP when interpreting and comparing model predictions, enabling researchers and clinicians to perform evaluations well and ultimately trust the resulting predictions [42].

In SHAP analysis, the important features identified include disease activity and medication duration. Regarding the high importance of medication duration features, we interpret this as reflecting the chronic nature of the disease. Patients with longer medication use may represent a more treatment-resistant subgroup, which influences the outcome. The difference in importance ranking of Das28-ESR between Gini importance and SHAP values was observed. Gini importance reflects role of a feature in building the model internally [43]. SHAP values measure the average marginal contribution of a feature to the final prediction. The 2 metrics are complementary, not contradictory.

A notable limitation of this study is the substantial missingness (53.2%) in the baseline ACPA (anti-CCP) data. While our comprehensive sensitivity analyses (comparing mean, median, KNN, and multiple imputation) confirmed that the overall model performance and conclusion remained robust, addressing such high missingness inherently introduces uncertainty. To further validate our findings, a subgroup analysis using 3-category ACPA classification was conducted, which consistently affirmed the model’s robustness and the variable’s predictive direction. ML algorithms (SVM, RF, AdaBoost, and DNN) are robust to minor numerical perturbations. From an empirical perspective, the presence of missing data did not substantially affect the main conclusions of our study. Model performance was consistent across imputation methods, indicating that our findings possess good robustness.

Some potential limitations should be considered when interpreting our study’s results. First, although all medications were classified based on their mechanisms, a highly detailed classification of DMARDs should be established to enhance the precision of predictions, given the numerous drugs involved with distinct mechanisms. Second, the collection of data from 5 distinct hospitals introduced inconsistencies into EHR register systems. The internal disparity in disease activity between the training and external validation datasets explains the diminished accuracy observed during external validation. Differences in data distributions across hospitals likely contributed to low performance on external datasets because our data processing methods and algorithm adjustments were primarily optimized for the PKUPH dataset. Our findings may need further validation in non-Chinese populations and different healthcare systems. The potential for bias, particularly regarding gender and age, should be noted in consideration of the demographic skew of patients with RA. We emphasize that future studies should rigorously assess model fairness across these subgroups and establish a continuous, large-scale prospective registry to further validate the models. Finally, the lack of a definitive clinical method for predicting RA disease activity that could serve as a gold standard for this study also posed a challenge. While DAS28-ESR is a widely used assessment tool, it is important to simultaneously incorporate other disease activity measures such as clinical disease activity index (CDAI), simplified disease activity index (SDAI), or Boolean-based criteria. Existing research provides insufficient reference for comparison [17,19–21,44]. Future prospective studies will incorporate these stricter criteria to further validate the model’s robustness.

## Conclusion

Our study highlights the substantial efficacy of constructing ML models for predicting disease outcomes and recommending therapeutic choices across diverse hospital settings. Our developed predictive model is readily applicable to routine clinical situations and provides valuable support for precision medicine. The methodology employed in our model construction may contribute to the broad application of ML algorithms in the analysis of EHRs.

## Ethical Approval

Informed consent was obtained from all participants. This study was approved by the Ethics Committee of Peking University People’s Hospital (2020PHB300-01).

## Data Availability

The datasets are not publicly available. De-identified data are available from the corresponding authors on reasonable request.
