# Supplementary material for: Electronic Health Record-Based Machine Learning Model for Predicting Disease Activity in Patients with Rheumatoid Arthritis
Source: Health Data Sci. 2026 Jun 8;6:0461. doi: 10.34133/hds.0461 (PMC13243797; doi:10.34133/hds.0461)
Supplement: Supplementary 1 — Supplementary Text Tables S1 to S4 Figs. S1 to S3 [file hds.0461.f1.docx]

**Supplementary Materials**

# Introduction of models

**1. DNN Model**

Deep Neural Networks (DNN) are neural network models composed of an input layer, one or multiple hidden layers, and an output layer. By leveraging the hierarchical structure of multi-layer neural networks, DNN extracts meaningful information from data through layer-by-layer processing. The deep architecture enables DNN to perform multi-level abstraction of raw data, automatically capturing complex structural features from massive datasets. It solves linearly inseparable problems that single-layer perceptrons cannot address. The figure below illustrates the topology of a DNN with two hidden layers.


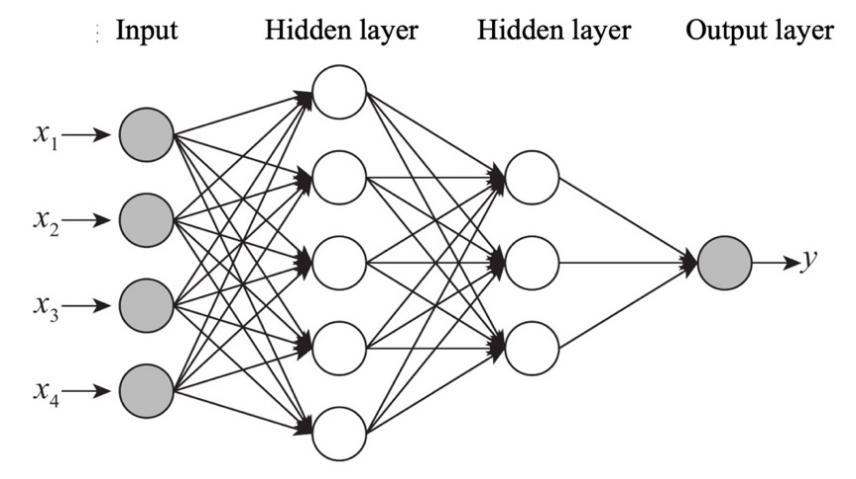


When used for classification, the number of input neurons corresponds to the dimensionality of the input signal, and the number of output neurons equals the number of classes. The loss function can be expressed as:


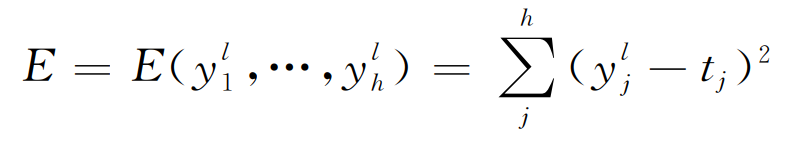


Here, the $l$ layer is the output layer, and $t_{j}$represents the expected output of the $j$neuron in the output layer. By computing the first-order partial derivative of the loss function, the weight update formula for the network is derived as:


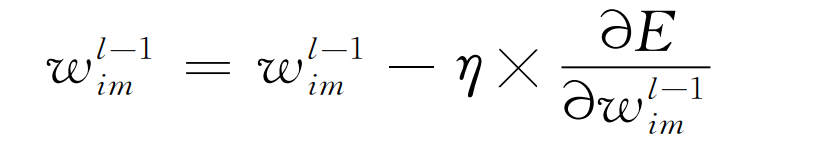


where $\eta$denotes the learning rate.

In the RA drug recovery prediction task, the DNN model outputs patient recovery status. The final layer uses the Sigmoid activation function, which maps input data to the interval (0,1), suitable for probabilistic outputs. A probability greater than 0.5 is labeled as 1 (indicating "no recovery after medication"), while a value ≤0.5 is labeled as 0 ("recovery predicted").

**2. SVM Model**


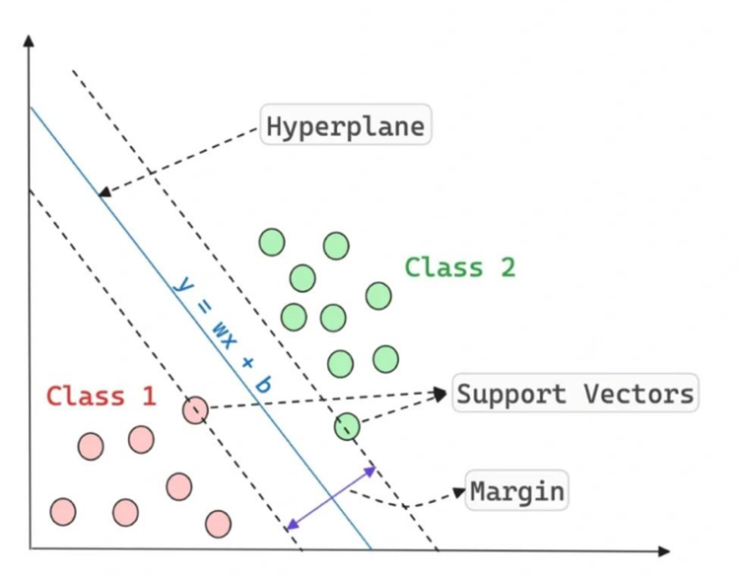


Support Vector Machine (SVM) is a supervised learning algorithm. Its core idea is to find an optimal hyperplane that separates data of different classes while maximizing the classification margin, as illustrated above. SVM handles linearly separable data (hard margin) or introduces slack variables and a penalty parameter C to accommodate linearly inseparable cases (soft margin). Nonlinear classification is achieved by mapping data to higher-dimensional space via kernel functions (e.g., Gaussian, polynomial). Implementation steps include:

1. Standardize data as preprocessing. 2. Choose a linear model (hard margin for strict separability; soft margin with penalty C to control errors) or kernel methods (e.g., Gaussian/polynomial kernels for nonlinear problems). 3. Define the optimization objective: minimize the norm of the weight vector for linear cases or solve the dual problem (Lagrange multipliers) for nonlinear cases. 4. Use quadratic programming to identify support vectors and construct the decision function (linear: inner product of weights; nonlinear: kernel function combinations). 5. Tune hyperparameters (e.g., regularization coefficient C, kernel parameter γ) via cross-validation to balance generalization and overfitting. In this study, the Gaussian kernel and C=10 were selected to predict RA patients’ medication recovery outcomes.

**3. RF Model**


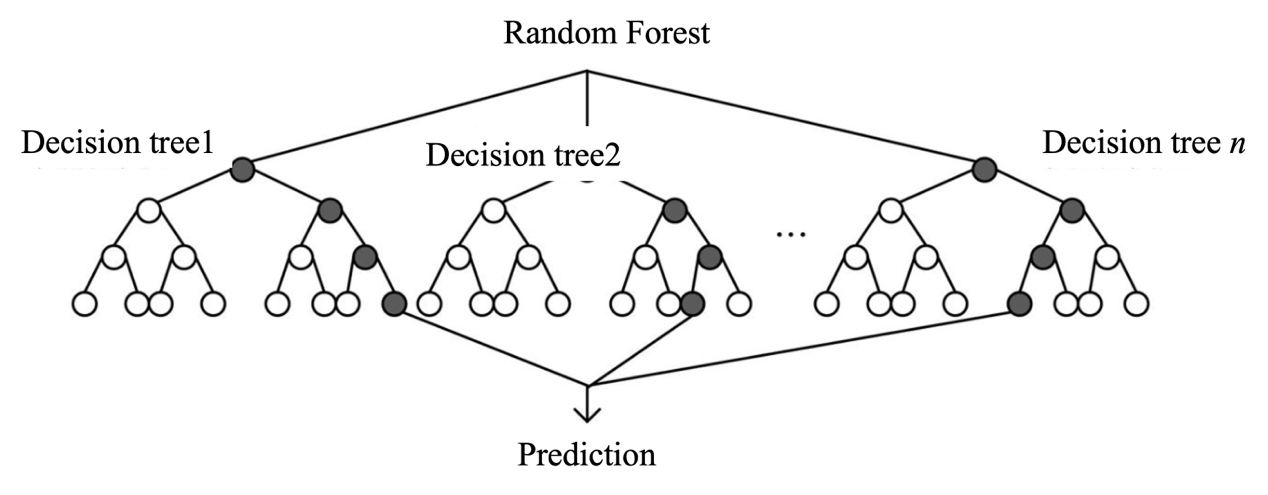


Random Forest (RF) is an ensemble learning algorithm that improves accuracy and robustness by aggregating predictions from multiple decision trees. Its workflow is as follows:

1. Bootstrap sampling: Generate multiple subsets from the original dataset via random sampling with replacement. Each subset trains a decision tree.

2. Feature randomness: At each node split, randomly select a subset of features (typically the square root of the total features) to compute the optimal split (using Gini index or information gain), ensuring diversity among trees.

3. Recursive splitting: Repeat the splitting process for child nodes until stopping conditions are met (e.g., minimum samples per node, maximum tree depth, or sufficient purity).

4. Aggregation: For classification, use majority voting; for regression, average the predictions.

The algorithm reduces overfitting through dual randomness (samples and features) and inherently supports parallel training.

**4. AdaBoost Model**


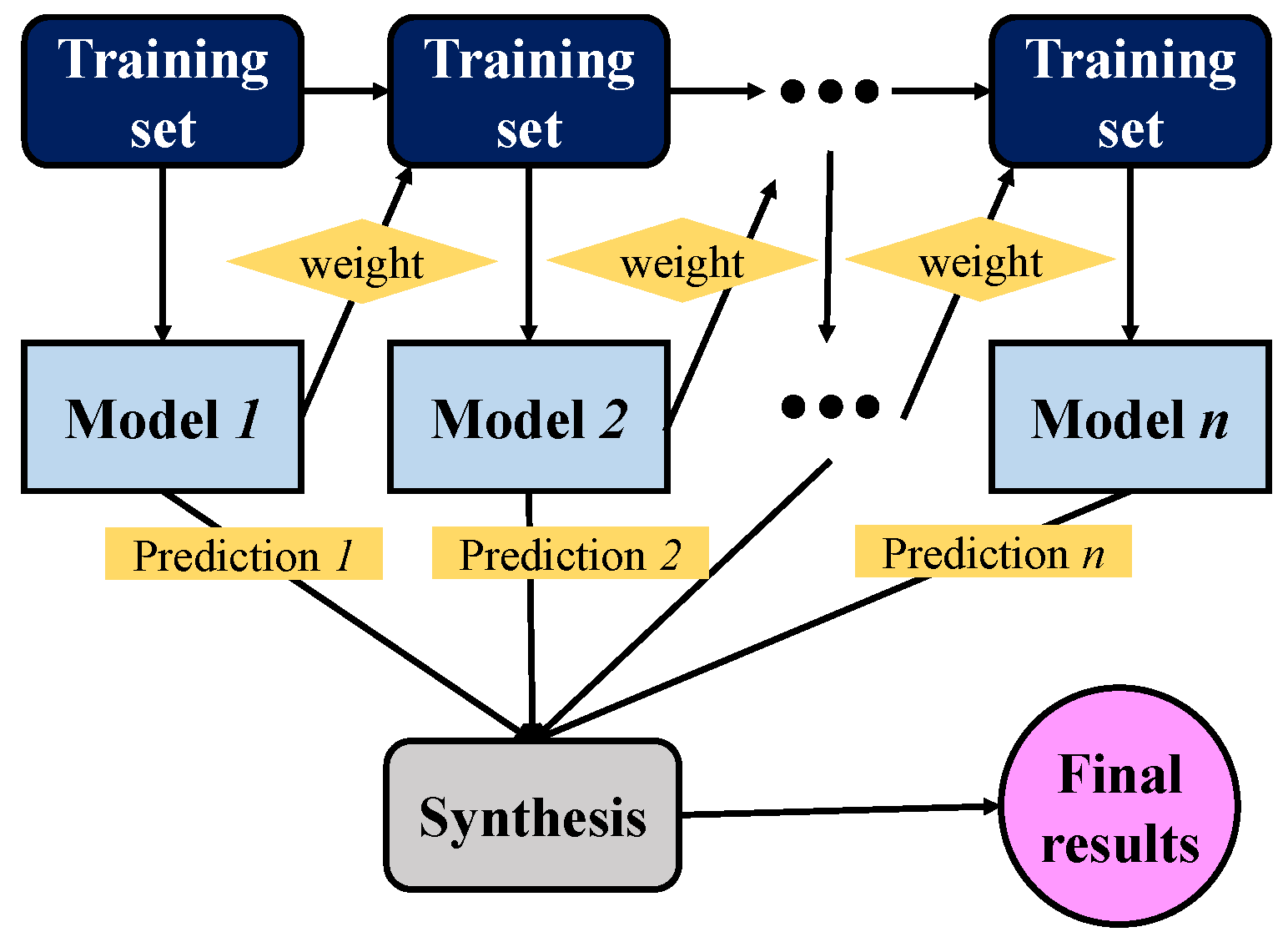


AdaBoost (Adaptive Boosting) is an ensemble learning algorithm that combines multiple weak classifiers into a strong classifier. The key idea is to iteratively train weak classifiers, adjusting sample weights to focus on misclassified examples in subsequent iterations. The workflow includes:

1. Initialize sample weights: Assign equal weight w1 = 1/N.

2. Iterative training:

Train a weak classifier on the current weighted data and compute its classification error. Assign a weight to the weak classifier based on its accuracy (higher accuracy → higher weight).

Update sample weights: Increase weights for misclassified samples and decrease for correctly classified ones.

3. Repeat teps 2-4 until the predefined number of weak classifiers is reached or error becomes zero.

4. Final prediction: Aggregate results via weighted voting, favoring decisions from high-accuracy weak classifiers.

**Table S1 Summary of missing data**

| **Variable** | **Total sample** | **Missing count** | **Missing (%)** |
| --- | --- | --- | --- |
| **ESR** | 5835 | 68 | 1.17 |
| **CRP** | 5835 | 909 | 15.58 |
| **RF** | 5835 | 958 | 16.42 |
| **Anti-CCP** | 5835 | 3104 | 53.2 |
| **Overall** | 23,340 | 5,039 | 21.59 |

Anti-CCP, anticyclic citrullinated peptide antibody; CRP, C-reactive protein; ESR, Erythrocyte Sedimentation Rate; RF, rheumatoid factor.

**Table S2 Sensitivity analysis of imputation methods**

| Method | Accuracy | AUROC | ESR coefficient | CRP coefficient | RF coefficient | Anti-CCP coefficient |
| --- | --- | --- | --- | --- | --- | --- |
| Mean imputation | 0.8672 | 0.7196 | 0.0363 | -0.0028 | 0 | 0.0001 |
| Median imputation | 0.8672 | 0.7218 | 0.0359 | -0.0012 | 0 | 0.0002 |
| KNN imputation | 0.8672 | 0.7194 | 0.0362 | -0.0023 | 0 | 0.0001 |
| Multiple imputation  (IterativeImputer) | 0.8672 | 0.7227 | 0.0354 | 0 | 0 | 0.0001 |
| Three-category ACPA classification | 0.8672 | 0.7321 | 0.0327 | 0 | 0 | 0.0001 |

Anti-CCP, anticyclic citrullinated peptide antibody; AUROC, the area under the receiver operating characteristic curve; CRP, C-reactive protein; ESR, Erythrocyte Sedimentation Rate; KNN K-Nearest Neighbors; RF, rheumatoid factor.

**Table S3. Parameters of different models**

| Algorithm | Parameters |
| --- | --- |
| DNN | Dense (units=128, activation='relu') first layer  Dense (units=64, activation='relu') second layer  Dense (units=32, activation='relu') third layer  Dense (units=1, activation='sigmoid') fourth layer  optimizer="adam"  loss='binary_crossentropy'  epochs=500, batch_size=256  verbose=1 |
| SVM | C=0.1  gamma=0.01  kernel="rbf" |
| AdaBoost | n_estimators=90, random_state=40, learning_rate=0.8 |
| RF | n_estimators=80, random_state=60, criterion="gini" |

The specific parameters of each model were displayed. DNN= deep neural network; SVM=support vector machine; AdaBoost= adaptive boosting; RF= random forest.

# Table S4. Accuracy of different machine learning models for secondary outcome.

|  | Accuracy (%) |
| --- | --- |
| DNN | 68.6 |
| AdaBoost | 59.5 |
| RF | 65.7 |

Overall accuracy of different machine learning models for secondary outcome prediction were displayed. The results were attained from tests in the multicenter cohort. DNN= deep neural network; AdaBoost= adaptive boosting; RF= random forest.


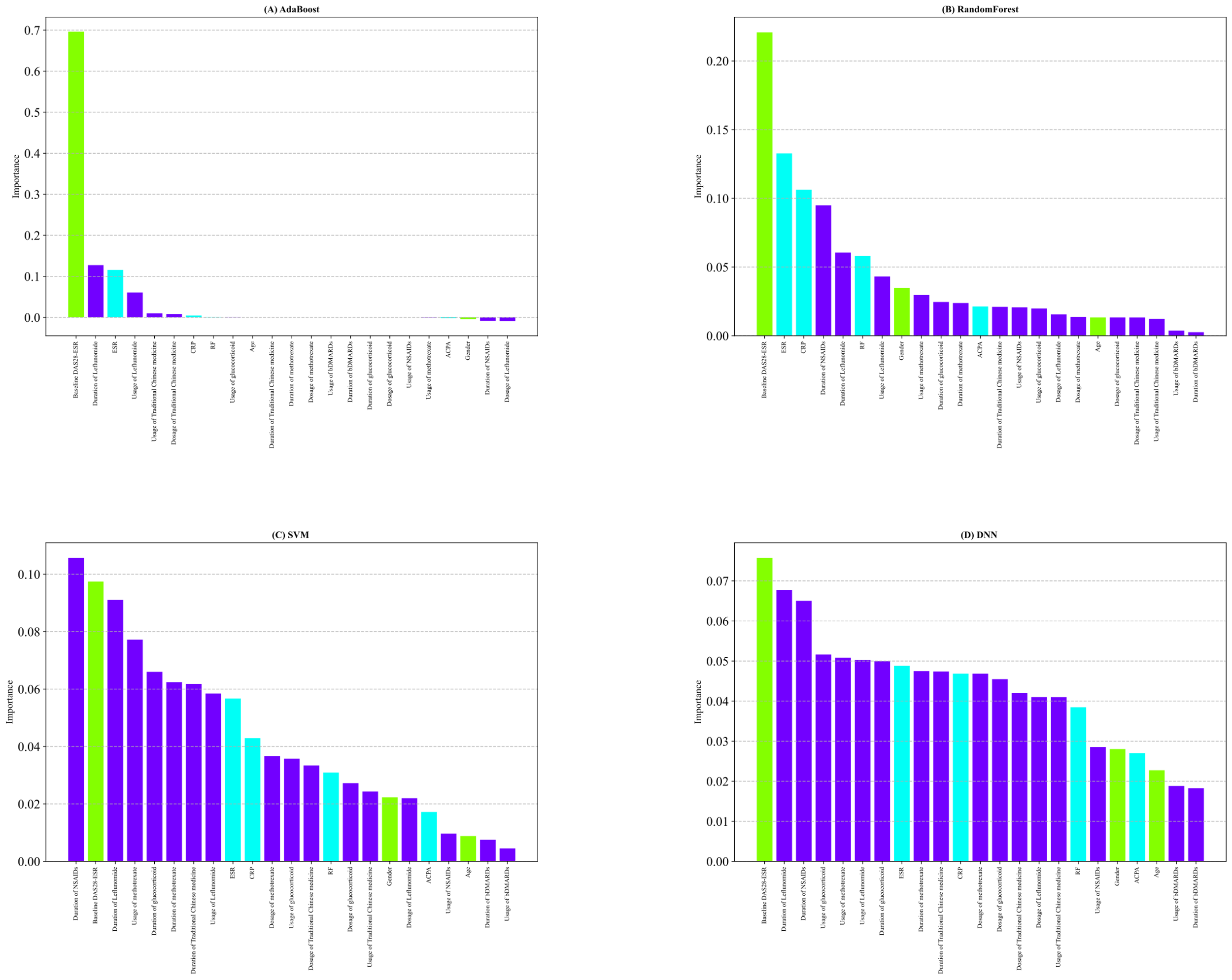


# Fig. S1. The importance of feature values of each machine learning model.

The X-axis represents the feature values. The Y-axis represents the importance score of features calculated by the Gini importance in AdaBoost (a) and RF (b) model, and permutation importance in SVM (c) and DNN (d) model. The features in every model are divided into different categories, represented by multiple colors of columns.

AdaBoost, adaptive boosting; ACPA= anti-citrullinated protein antibodies; CRP, C-reactive protein; DAS28, disease activity score using 28 joint counts; DNN, deep neural network; ESR, erythrocyte sedimentation rate; NSAIDs, nonsteroidal anti-inflammatory drugs; rhTNFR-Fc, recombinant human tumor necrosis factor receptor-Fc fusion protein; RF, random forest; RF, rheumatoid factors; SVM, support vector machine.


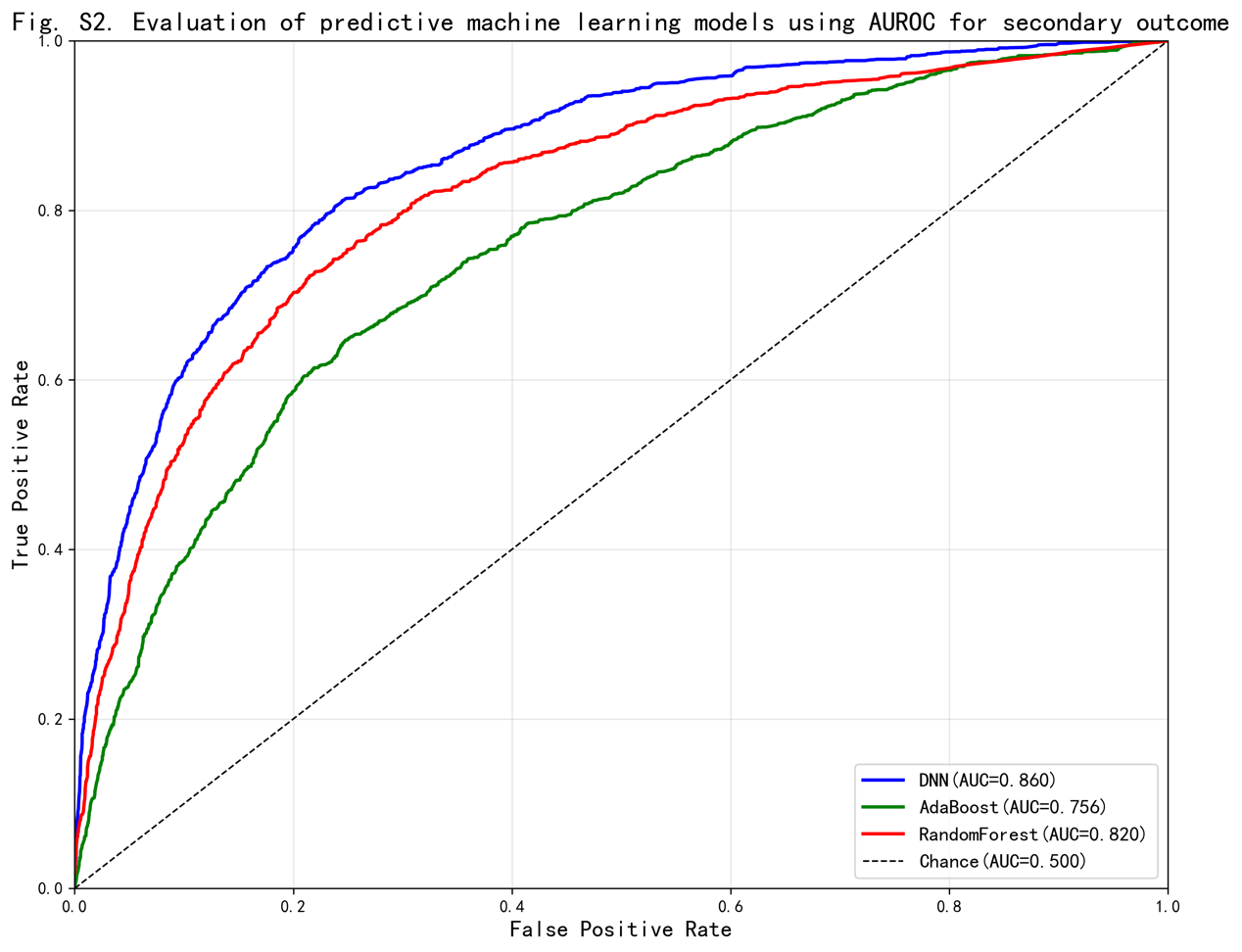


# Fig. S2. Evaluation of predictive machine learning models using AUROC for secondary outcome.

AUROC**,** the area under the receiver operating characteristic curve

AdaBoost, adaptive boosting; DNN=deep neural network.

# Fig. S3. The important ranking of variables according to the SHAP value.

SVM (A), DNN (B), RF (C) and AdaBoost (D) model
